# Supplementary material for: Development of One-Step Non-Solvent Extraction and Sensitive UHPLC-MS/MS Method for Assessment of N-(n-Butyl) Thiophosphoric Triamide (NBPT) and N-(n-Butyl) Phosphoric Triamide (NBPTo) in Milk
Source: Molecules. 2021 May 13;26(10):2890. doi: 10.3390/molecules26102890 (PMC8153019; doi:10.3390/molecules26102890)
Supplement: Supplementary file 1 [file molecules-26-02890-s001.zip › molecules-1201820-supplementary.pdf]

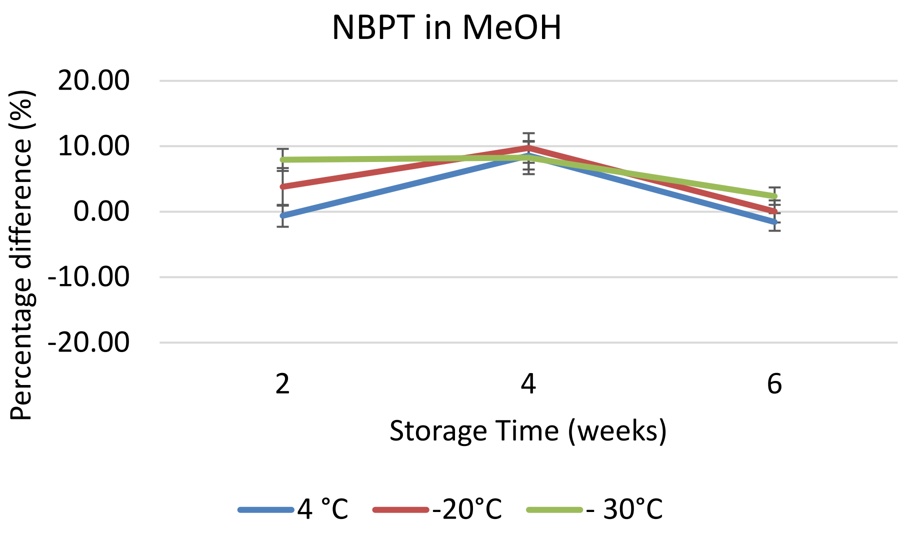

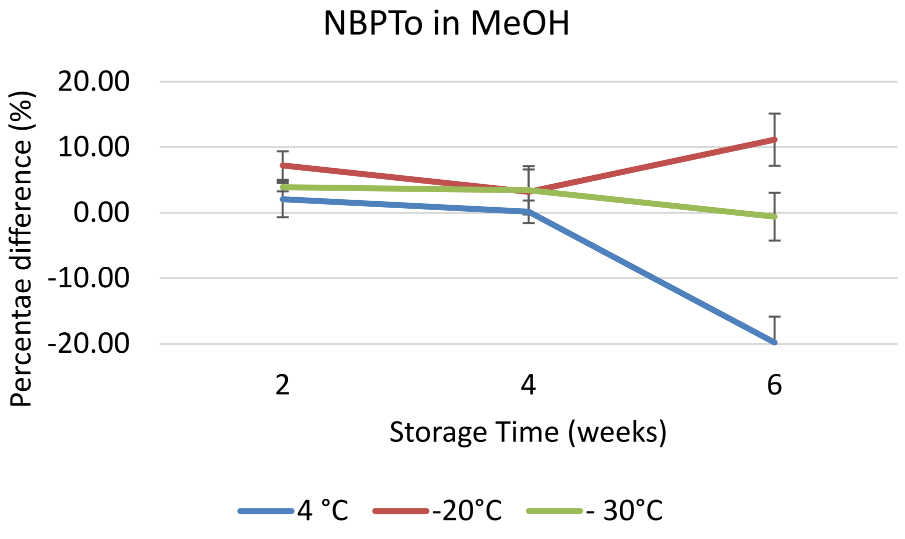

Supplementary Figure S1: Stability of NBPT and NBPTo, (1 mg kg^{-1}, respectively) stored at +4, -20 and -30 °C in MeOH for 6 weeks. Results indicate that NBPT residues were most stable in MeOH at all temperatures. NBPTo was also stable in MeOH at all temperatures tested except at +4 °C where it was stable for less than 6 weeks. Values are means ± RSD of 3 replicate samples.
